# Exploring spatial heterogeneity of PD-L1 and c-MET expression in oral squamous cell carcinoma

**DOI:** 10.1007/s10006-026-01638-1

**Published:** 2026-09-16

**Authors:** Cornelius Busse, Marius Hörner, Babak Saravi, Lukas Haug, Sebastian Gubik, Alexander Kübler, Urs Müller-Richter, Andreas Rosenwald, Stefan Hartmann

**Affiliations:** 1 Department of Oral and Maxillofacial Plastic Surgery and Head and Neck Surgery, University Hospital Würzburg, Pleicherwall 2, 97070 Würzburg, Germany; 2 Department of Oral, Maxillofacial and Facial Plastic Surgery, Medical Faculty, University Hospital Düsseldorf, Heinrich-Heine-University Düsseldorf, 40225 Düsseldorf, Germany; 3 Dr. Senckenberg Institute of Pathology, University Hospital Frankfurt, Goethe University Frankfurt, Theodor-Stern-Kai 7, 60590 Frankfurt am Main, Germany; 4 Institute of Pathology, University of Würzburg, Würzburg, Germany

**Keywords:** Oral squamous cell carcinoma, PD-L1, c-MET, spatial heterogeneity, lymph node metastasis, biomarkers

## Abstract

**Background:**

PD-L1 expression is an established predictive biomarker for immune checkpoint inhibitor therapy in recurrent or metastatic head and neck squamous cell carcinoma (HNSCC), including oral squamous cell carcinoma (OSCC). However, the spatial heterogeneity of PD-L1 and additional biomarkers such as c-MET between primary tumors and lymph node metastases remains insufficiently characterized. Given the potential for compartment-specific biomarker differences between primary tumors and metastatic lesions, we investigated PD-L1 and c-MET expression patterns in matched primary tumors and lymph node metastases of OSCC patients.

**Methods:**

In this retrospective cohort study, PD-L1 expression (Tumor Proportion Score [TPS], Combined Positive Score [CPS]) and c-MET expression (H-score, percentage-based scoring) were analyzed by immunohistochemistry in primary tumors from 59 OSCC patients. Matched primary tumor–lymph node metastasis pairs were available for 24 patients and served as the basis for the paired spatial heterogeneity analyses. Biomarker concordance between primary tumors and metastases as well as associations with clinicopathologic parameters were evaluated.

**Results:**

Both PD-L1 and c-MET were frequently expressed in primary tumors but showed significantly lower expression levels in matched lymph node metastases. Higher PD-L1 expression in primary tumors was associated with nodal metastasis in exploratory analyses, whereas c-MET expression showed no significant association with nodal status. No significant correlation between PD-L1 and c-MET expression was observed. Furthermore, discordance of PD-L1 CPS status between primary tumors and lymph node metastases resulted in different biomarker classifications in a substantial subset of matched cases. In exploratory analyses, biomarker expression and primary tumor–lymph node discordance were not significantly associated with recurrence, although the limited number of events precludes definitive conclusions.

**Conclusions:**

Our findings demonstrate pronounced spatial heterogeneity of PD-L1 and c-MET expression in OSCC and reveal compartment-specific biomarker expression patterns between primary tumors and lymph node metastases. The observed discordance between tumor compartments warrants further investigation in larger prospective studies to determine its biological and potential clinical relevance for biomarker assessment in OSCC.

**Supplementary Information:**

The online version contains supplementary material available at https://doi.org/10.1007/s10006-026-01638-1.

## Introduction

Head and neck squamous cell carcinoma (HNSCC) ranks among the most common malignancies worldwide, with approximately 890,000 new cases diagnosed annually [1]. Oral squamous cell carcinoma (OSCC) represents the most frequent subtype of HNSCC and remains associated with high recurrence rates and poor clinical outcome despite multimodal treatment approaches [1, 2]. In recent years, immunotherapeutic strategies have increasingly moved into the focus of treatment for head and neck cancers, underscoring the need for reliable biomarkers that may guide patient stratification and therapeutic decision-making [3]. In particular, the KEYNOTE-048 and KEYNOTE-689 trials established PD-L1 expression as a clinically relevant biomarker in recurrent/metastatic and curative treatment settings [3, 4].

Programmed death-ligand 1 (PD-L1) represents one of the most established predictive biomarkers in HNSCC and is commonly assessed using the Combined Positive Score (CPS), which incorporates PD-L1 expression in both tumor and immune cells [5]. While PD-L1 has predictive value for response to immune checkpoint inhibition, increasing evidence suggests that its expression may vary substantially between primary tumors and metastatic lesions, raising concerns regarding spatial heterogeneity and biomarker reliability [5].

In addition to PD-L1, the hepatocyte growth factor (HGF)/MET signaling pathway has emerged as an important regulator of OSCC biology. c-MET overexpression has been associated with tumor progression, invasion, and poor clinical outcome in OSCC [6, 7]. Experimental studies further suggest interactions between c-MET signaling and tumor immune regulation, including modulation of PD-L1 expression [8, 9].

However, the spatial distribution of PD-L1 and c-MET expression between primary tumors and lymph node metastases in OSCC remains insufficiently characterized. In particular, it is unclear whether biomarker status remains stable across tumor compartments and to what extent metastatic lesions differ biologically from the corresponding primary tumor.

Therefore, we retrospectively analyzed PD-L1 and c-MET expression in primary tumors and matched lymph node metastases of OSCC patients using multiple immunohistochemical scoring systems (TPS, CPS, and H-score). As an exploratory analysis, we sought to investigate spatial biomarker heterogeneity, evaluate concordance between tumor compartments, and explore potential associations between PD-L1 and c-MET expression.

## Materials and methods

### Study population

We retrospectively included 59 patients diagnosed with OSCC and treated at the Department of Oral and Maxillofacial Plastic Surgery and Head and Neck Surgery at the University Hospital in Würzburg between December 2009 and May 2011. Eligible cases had available tumor tissue or lymph node specimens for immunohistochemical analysis and sufficient follow-up data. Clinical information, including age, sex, tumor site, TNM classification, therapy type, and resection margin (R) status, was retrieved from institutional records. Patients were followed up until recurrence or last available contact.

### Human Ethics and Consent to Participate

The study was conducted in accordance with the Declaration of Helsinki and approved by the Ethics Committee of the University of Würzburg, Germany (Approval No. 20191129 02). Due to the retrospective nature of the study and the use of anonymized archival tissue specimens, the requirement for informed consent was waived by the Ethics Committee.

### Immunohistochemistry

PD-L1 expression was assessed using tumor proportion score (TPS) and combined positive score (CPS) and c-MET expression was evaluated by H-Score.

Immunohistochemical staining for c-MET was performed using the ultraView Universal DAB Detection Kit on the BenchMark Ultra IHC/ISH automated staining platform (Ventana, Roche, Tucson, AZ, USA). c-MET expression was evaluated with the CONFIRM anti-total c-MET (SP44) rabbit monoclonal primary antibody (Ventana, Roche; dilution 1:1000).

PD-L1 expression was assessed using the rabbit monoclonal antibody clone 28 − 8 (Cell Marque, Rocklin, CA, USA; dilution 1:1000) on the Leica BOND-III automated staining platform (Leica Biosystems, Nussloch, Germany). Of note, the FDA-approved companion diagnostic for pembrolizumab in HNSCC uses clone 22C3 (PD-L1 IHC 22C3 pharmDx); however, previous studies have demonstrated high concordance between 28 − 8 and 22C3 assays in HNSCC [10].

### Assessment of PD-L1 Expression (TPS/CPS Evaluation)

PD-L1 expression was assessed using both the tumor proportion score (TPS) and the combined positive score (CPS). TPS was calculated as the percentage of PD-L1–positive tumor cells among all viable tumor cells, whereas CPS was defined as the number of PD-L1–positive tumor cells, lymphocytes, and macrophages divided by the total number of viable tumor cells, multiplied by 100. Regions of Interest (ROI) with fewer than 100 viable tumor cells were excluded from the analysis as previous reported [10].

For descriptive and statistical analyses, CPS values were recorded as originally assessed and were not capped at 100. This approach was chosen to preserve the full quantitative range of PD-L1 expression. Clinically relevant categorical analyses were subsequently performed using established CPS thresholds (≥ 1, ≥ 10, and ≥ 20).

### Assessment of c-MET Expression (H-Score)

c-MET expression was assessed using the H-Score, calculated based on five predefined regions of interest (ROI), each containing more than 100 viable tumor cells and collectively representing the overall FFPE tissue section. The H-Score is a validated semiquantitative metric defined as:

### H-Score = (1 × percentage of weakly stained cells) + (2 × percentage of moderately stained cells) + (3 × percentage of strongly stained cells)

The mean H-Score across the five ROIs was then determined, yielding a final score ranging from 0 to 300. This method enables a semiquantitative assessment of both the proportion of positively stained viable tumor cells and the corresponding staining intensity.

For exploratory categorical analyses, H-score thresholds of > 50, >75, and > 100 were applied. As no universally accepted or clinically validated c-MET cutoffs currently exist for OSCC, these thresholds were used to facilitate comparison across expression levels and to assess biomarker concordance between tumor compartments. The resulting categorizations should therefore be interpreted as exploratory rather than clinically validated classifications.

PD-L1 and c-MET expression were evaluated jointly by an experienced pathologist (L.H.) and an experienced oral and maxillofacial surgeon (C.B.). All cases were reviewed in consensus and a final score was assigned accordingly. Formal assessment of interobserver variability was not performed. Owing to the evident morphological differences between primary tumor and lymph node tissue, blinding to tumor compartment (primary versus metastasis) was not feasible; however, the observers were blinded to clinical outcome data during biomarker assessment.

### Statistical analysis

Data were analyzed using Python 3.12 with pandas, scipy, and statsmodels libraries. Descriptive statistics included median with interquartile range (IQR) for continuous variables and frequencies with percentages for categorical variables. Normality was assessed using the Shapiro–Wilk test; as biomarker expression data were non-normally distributed, non-parametric tests were applied throughout. For paired comparison of biomarker expression between primary tumors and matched lymph node metastases, the Wilcoxon signed-rank test was used. The median of within-patient paired differences (Δ) was reported rather than the difference of medians. Unpaired group comparisons (e.g., N0 vs. N+) were performed using the Mann–Whitney U test for continuous variables and Fisher’s exact test for categorical variables. Odds ratios (OR) with direction indicating the odds of marker positivity in N+ versus N0 patients were calculated. Correlations between PD-L1 and c-MET expression were assessed using Spearman’s rank correlation coefficient (ρ). Concordance of biomarker status between primary tumor and lymph node metastases was evaluated using Cohen’s kappa (κ), with interpretation as follows: <0 less than chance agreement; 0.01–0.20 slight; 0.21–0.40 fair; 0.41–0.60 moderate; 0.61–0.80 substantial; 0.81–1.00 almost perfect agreement. c-MET expression data were available for 58 of 59 primary tumors; one patient was excluded from c-MET analyses due to insufficient tumor tissue for immunohistochemical evaluation. This patient had matched lymph node metastases, resulting in *n* = 23 for paired c-MET comparisons versus *n* = 24 for paired PD-L1 comparisons. A two-sided p-value < 0.05 was considered statistically significant. No adjustment for multiple comparisons was performed given the exploratory nature of the study.

Follow-up data were available for locoregional recurrence, which was recorded together with the date of recurrence; patients without recurrence were censored at the date of last documented contact. Recurrence-free survival (RFS) was defined as the interval from diagnosis to first locoregional recurrence or censoring and was estimated using the Kaplan–Meier method (Python lifelines package); groups were compared with the log-rank test, and univariable hazard ratios (HR) with 95% confidence intervals (CI) were derived from Cox proportional-hazards models. Given the small number of events, these outcome analyses were considered exploratory and no multivariable modeling was performed. A conventional disease-free survival endpoint, overall survival, and distant-metastasis-free survival could not be analyzed, as distant recurrence, vital status, and distant-metastasis staging were not systematically available for this historical cohort (distant metastasis was classified as cMX in all patients).

## Results

### Patient and Tumor Characteristics

A total of 59 patients with histologically confirmed oral squamous cell carcinoma (OSCC) and evaluable tumor tissue were included in the study. Matched lymph node (LN) metastases were available for paired analysis in 24 patients (40.7%). Detailed clinicopathological characteristics of the cohort are summarized in Table 1. The mean age at diagnosis was 61.7 ± 11.8 years, with a male predominance (69.5% male, 30.5% female). Regarding adjuvant treatment, the majority of patients received radio-chemotherapy (47.5%) or radiotherapy alone (27.1%), while 23.7% underwent surgery followed by surveillance only and one patient (1.7%) received chemotherapy alone. Most patients presented with early-stage disease (T1–T2, 74.6%), while advanced tumors (T3–T4) were observed in 25.4%. Pathological lymph node involvement was present in 25 patients (42.4%), whereas 34 patients (57.6%) were classified as node-negative (N0). Tumor staging was assessed according to the 7th edition of the TNM classification system [11]. Histological grading predominantly revealed moderately differentiated tumors (G2, 66.1%), with well-differentiated (G1) and poorly differentiated (G3) carcinomas accounting for 5.1% and 25.4%, respectively. Complete tumor resection with negative margins (R0) was achieved in 79.7% of cases.

**Table 1:** Baseline patient and tumor characteristics of the study cohort

Characteristic	*n*	%
Age, years (mean ± SD)	61.7 ± 11.8	**-**
Sex		
Male	41	69.5
Female	18	30.5
T-Stage		
pT1	17	28.8
pT2	27	45.8
pT3	4	6.8
pT4a	11	18.6
Nodal Status		
N0	34	57.6
N+	25	42.4
Histological Grade		
G1 (well differentiated)	3	5.1
G2 (moderately differentiated)	39	66.1
G3 (poorly differentiated)	15	25.4
Resection Margin		
R0 (complete)	47	79.7
R1 (microscopic residual)	5	8.5
RX (cannot be assessed)	5	8.5
Adjuvant Treatment		
Surveillance only	14	23.7
Radiotherapy	16	27.1
Radio-chemotherapy	28	47.5
Chemotherapy alone	1	1.7
Invasion Status		
Lymphovascular invasion (L1)	10	17.5
Venous invasion (V1)	3	5.2
Perineural invasion (Pn1)	9	15.8
Matched LN metastases available	24	40.7

Abbreviations: *LN* lymph node, *SD* standard deviation. Percentages may not sum to 100% due to missing values

### PD-L1 and c-MET Expression in Primary Tumors and Lymph Node Metastases

Immunohistochemical analyses demonstrated substantial PD-L1 and c-MET expression in the primary tumor compartment (Table 2). PD-L1 expression, assessed by the Tumor Proportion Score (TPS), showed a median of 5.0% (IQR 1.0–40.0), with a wide range from 0% to 100%. The Combined Positive Score (CPS) indicated even broader expression, with a median of 15.0 (IQR 5.0–77.5; range 0–150).

**Table 2:** PD-L1 and c-MET expression in primary tumors and lymph node metastases Clinically Relevant Cutoffs:

Variable	Compartment	*n*	Median (IQR)	Mean ± SD	Range
PD-L1 TPS (%)	Primary Tumor	59	5.0 (1.0–40.0)	23.2 ± 32.0	0–100
	LN Metastasis	24	1.0 (1.0–6.2)	10.1 ± 21.7	0–100
PD-L1 CPS	Primary Tumor	59	15.0 (5.0–77.5)	39.1 ± 42.9	0–150
	LN Metastasis	24	7.5 (5.0–50.0)	28.1 ± 33.6	0–100
c-MET H-Score	Primary Tumor	58	92.1 (76.1–102.9)	87.8 ± 36.2	0–190
	LN Metastasis	24	70.9 (19.3–89.1)	58.3 ± 39.4	0–133
c-MET % positive	Primary Tumor	58	78.7 (64.1–87.2)	72.0 ± 24.0	0–100
	LN Metastasis	24	60.6 (18.9–82.7)	52.6 ± 34.0	0–95
Cutoff	Primary Tumor *n*/*N* (%)	LN Metastasis *n*/*N* (%)			
PD-L1 TPS ≥ 1%	52/59 (88.1%)	21/24 (87.5%)			
PD-L1 CPS ≥ 1	52/59 (88.1%)	21/24 (87.5%)			
PD-L1 CPS ≥ 10	40/59 (67.8%)	12/24 (50.0%)			
PD-L1 CPS ≥ 20	29/59 (49.2%)	10/24 (41.7%)			
c-MET H-Score > 50	50/58 (86.2%)	16/24 (66.7%)			
c-MET H-Score > 75	45/58 (77.6%)	10/24 (41.7%)			
c-MET H-Score > 100	20/58 (34.5%)	2/24 (8.3%)			

Abbreviations: *CPS* Combined Positive Score, *IQR* interquartile range, *LN* lymph node, *SD* standard deviation, *TPS* Tumor Proportion Score

c-MET expression in primary tumors was likewise high, with a median H-Score of 92.1 (IQR 76.1–102.9; range 0–190). Overall, 77.6% of tumors showed c-MET overexpression (H-Score > 75), and 34.5% exceeded an H-Score of 100.

In contrast, biomarker expression in lymph node metastases was consistently lower. PD-L1 TPS exhibited a median of 1.0% (IQR 1.0–6.2), and the median c-MET H-Score decreased to 70.9 (IQR 19.3–89.1). Accordingly, the proportion of samples exceeding clinically relevant thresholds was reduced in lymph node metastases: CPS ≥ 20 was observed in 41.7% compared with 49.2% of primary tumors, and c-MET overexpression (H-Score > 75) was present in only 41.7% versus 77.6% of primary tumors (Suppl. Figure 1).

### Paired Comparison Reveals Significant Downregulation in Lymph Node Metastases

**Fig. 1:**
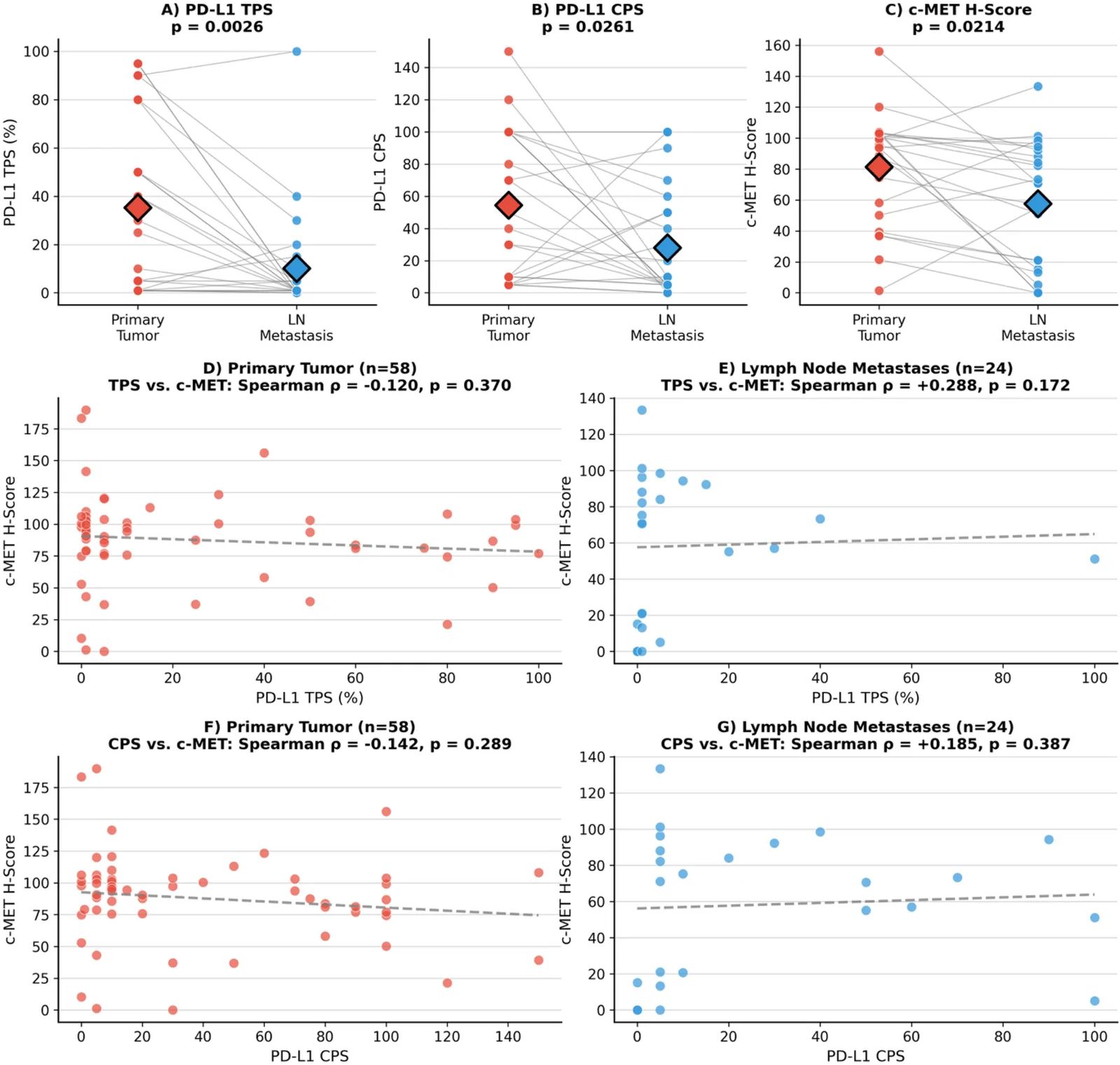
Spatial differences in PD-L1 and c-MET expression and association with nodal status in OSCC. (A–C) Paired comparison of biomarker expression between matched primary tumors and lymph node (LN) metastases: PD-L1 TPS (A), PD-L1 CPS (B), and c-MET H-score (C). Red indicates primary tumors and blue indicates LN metastases; gray lines connect paired samples. Diamonds represent mean values. Wilcoxon signed-rank test. All markers were significantly higher in primary tumors (TPS: p = 0.003; CPS: p = 0.026; c-MET: p = 0.021). (D–E) Correlation between PD-L1 TPS and c-MET H-score in primary tumors (D, n = 58) and LN metastases (E, n = 24). (F–G) Correlation between PD-L1 CPS and c-MET H-score in primary tumors (F, n = 58) and LN metastases (G, n = 24). Dashed lines indicate linear regression; Spearman ρ and p-values are shown. No significant correlations were observed

The central finding of this study emerged from the paired analysis of matched primary tumor and lymph node metastases specimens (Suppl. Table 1, Fig. 1A**- C**). Significant decreases were observed for PD-L1 TPS, PD-L1 CPS, and c-MET H-score, while c-MET % positive showed a non-significant trend toward lower values in LN metastases.

TPS decreased significantly from a median of 27.5% in primary tumors to 1.0% in matched LN metastases (Δ = +16.5, *p* = 0.003). This downregulation was observed in 67% of patients (16/24), while only 17% (4/24) showed increased TPS in metastases. Similarly, CPS demonstrated significant reduction from a median of 45.0 in primary tumors to 7.5 in LN metastases (Δ = +17.5, *p* = 0.026), with 71% of patients showing decreased expression in metastatic sites. c-MET expression followed the same pattern, with H-Score decreasing from a median of 93.7 in primary tumors to 70.7 in LN metastases (Δ = +18.6, *p* = 0.021). Remarkably, 78% of patients (18/23) demonstrated lower c-MET expression in their LN metastases compared to the primary tumor.

### Correlation between PD-L1 and c-MET Expression

We investigated the potential co-expression of PD-L1 and c-MET, which could have therapeutic implications for combined targeting strategies (Table 3; Fig. 1D-G). Spearman correlation analysis revealed no significant correlation between PD-L1 and c-MET expression in primary tumors (TPS vs. H-Score: ρ = -0.120, *p* = 0.370, Fig. 1D, E; CPS vs. H-Score: ρ = -0.142, *p* = 0.289, Fig. 1F, G). A trend toward positive correlation was observed in lymph node metastases, with TPS vs. c-MET percentage showing the strongest association (ρ = +0.377, *p* = 0.069). However, this trend did not reach statistical significance. Binary analysis using predefined categorical thresholds (CPS ≥ 20 and H-Score > 75) also failed to demonstrate significant association in either compartment (Fisher’s exact test: primary tumor OR = 0.55, *p* = 0.530; LN metastases OR = 0.89, *p* = 1.000). In this exploratory analysis, PD-L1 and c-MET expression were not significantly correlated; however, the study was not powered to exclude a modest association, particularly in lymph node metastases.

**Table 3:** Correlation between PD-L1 and c-MET expression. A. Spearman Correlation Analysis

Compartment	Comparison	*n*	Spearman ρ	*p*-value		
Primary Tumor	TPS vs. c-MET H-Score	58	−0.120	0.370		
	CPS vs. c-MET H-Score	58	−0.142	0.289		
	TPS vs. c-MET % positive	58	−0.069	0.608		
	CPS vs. c-MET % positive	58	−0.071	0.598		
LN Metastasis	TPS vs. c-MET H-Score	24	+ 0.288	0.172		
	CPS vs. c-MET H-Score	24	+ 0.185	0.387		
	TPS vs. c-MET % positive	24	+ 0.377	0.069		
	CPS vs. c-MET % positive	24	+ 0.310	0.140		
B. Binary Analysis (CPS ≥ 20 vs. H-Score > 75)						
Compartment	CPS ≥ 20 / H > 75	CPS ≥ 20 / H ≤ 75	CPS < 20 / H > 75	CPS < 20 / H ≤ 75	OR	*p*-value
Primary Tumor	21	8	24	5	0.55	0.530
LN Metastasis	4	6	6	8	0.89	1.000

Spearman rank correlation and Fisher's exact test. No significant correlations were observed. Abbreviations: *CPS* Combined Positive Score, *LN* lymph node, *OR* odds ratio, *TPS* Tumor Proportion Score

### Association of Primary Tumor Expression with Nodal Metastasis

**Fig. 2:**
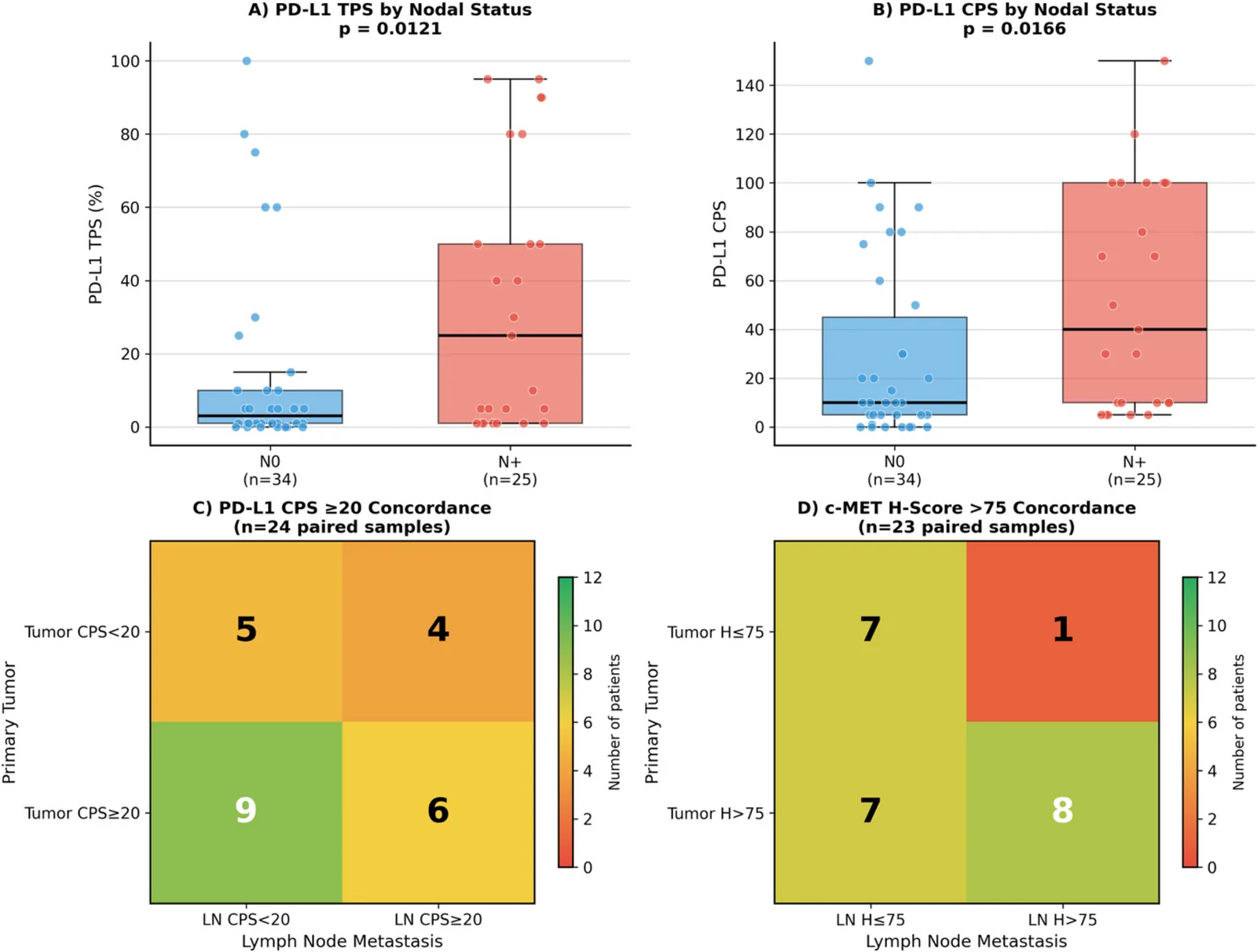
Association of PD-L1 expression with nodal status and biomarker concordance between primary tumors and lymph node metastases. (A–B) Association of PD-L1 expression in primary tumors with nodal status. Distributions of (A) PD-L1 tumor proportion score (TPS) and (B) PD-L1 combined positive score (CPS) in patients without (N0, blue) and with (N+, red) lymph node metastases. Box plots show median and interquartile range with individual data points overlaid. P-values were calculated using the Mann–Whitney U test. Both PD-L1 TPS (p = 0.012) and CPS (p = 0.017) were significantly higher in N+ patients. (C–D) Concordance matrices of biomarker status between matched primary tumors and lymph node (LN) metastases. (C) PD-L1 CPS using the clinically relevant cutoff of CPS ≥20 (KEYNOTE-048) in 24 paired samples and (D) c-MET expression using an H-Score >75 cutoff in 23 paired samples. Numbers indicate patient counts per category. Color gradients illustrate concordant (diagonal) versus discordant (off-diagonal) classification. For PD-L1 CPS ≥20, only 45.8% of patients showed concordant status, with 37.5% displaying CPS ≥20 in the primary tumor but CPS <20 in the corresponding LN metastasis

Given the clinical importance of nodal status in OSCC prognosis and treatment planning, we examined whether biomarker expression in the primary tumor was associated with the presence of lymph node metastases (Table 4; Fig. 2A, B).

**Table 4:** Association of biomarker expression in primary tumor with nodal status. A. Continuous Variables (Mann-Whitney U Test)

Marker	N0 (*n* = 34)	N+ (*n* = 25)	*p*-value	
PD-L1 TPS (%)	3.0 (1.0–10.0)	25.0 (1.0–50.0)	0.012*	
PD-L1 CPS	10.0 (5.0–45.0)	40.0 (10.0–100.0)	0.017*	
c-MET H-Score*§*	90.5 (78.9–106.1)	93.6 (56.2–102.9)	0.453	
c-MET % positive*§*	81.1 (74.6–87.7)	72.8 (49.9–85.0)	0.132	
B. Categorical Variables (Fisher’s Exact Test)				
Cutoff	N0 *n*/*N* (%)	*N* + *n*/*N* (%)	OR	*p*-value
PD-L1 TPS ≥ 1%	27/34 (79.4%)	25/25 (100.0%)	NE‡	0.017*
PD-L1 CPS ≥ 20	14/34 (41.2%)	15/25 (60.0%)	2.14 (0.76–6.06)	0.192
c-MET H-Score > 75	29/34 (85.3%)	16/24 (66.7%)	0.35 (0.09–1.29)	0.118

*p<0.05. Values are median (interquartile range). Abbreviations: *CPS* Combined Positive Score, *OR* odds ratio, *TPS* Tumor Proportion Score. †OR indicates odds of marker positivity in N+ versus N0 patients; OR >1 indicates higher odds of positivity in patients with nodal metastases. ‡NE, not estimable due to complete separation (all N+ patients were TPS-positive). § c-MET analyses were based on 24 N+ patients (one N+ case was not evaluable for c-MET); the N0 group comprised 34 patients for all markers

PD-L1 expression in primary tumors was significantly higher in patients with nodal metastases (N+) compared to those without (N0). Median PD-L1 CPS was significantly elevated in N+ patients (40.0 vs. 10.0 in N0 patients, *p* = 0.017). Similarly, median PD-L1 TPS was higher in N+ patients (25.0% vs. 3.0% in N0 patients, *p* = 0.012). All N+ patients (100%) demonstrated PD-L1 positivity (TPS ≥ 1%), compared to 79.4% of N0 patients (*p* = 0.017). In contrast, c-MET expression showed no significant association with nodal status. Median H-Score was comparable between N0 (90.5) and N+ (93.6) patients (*p* = 0.453). These findings suggest an association between elevated PD-L1 expression and nodal status; however, causality and independence from other clinicopathological factors cannot be inferred from the present analysis.

### Discordance of Biomarker Status Between Primary Tumors and Lymph Node Metastases

An open question is whether biomarker assessment in primary tumor tissue reflects biomarker status in metastatic disease. We therefore evaluated the concordance of biomarker positivity between matched primary tumors and lymph node (LN) metastases using clinically relevant cutoffs (Table 5; Fig. 2C, D).

**Table 5:** Concordance of biomarker status between primary tumor and lymph node metastases

Cutoff	T+/LN+	T−/LN−	T+/LN−	T−/LN+	Concordance	κ
PD-L1 TPS ≥ 1%	21	0	3	0	87.5%	0.00
PD-L1 CPS ≥ 1	21	0	3	0	87.5%	0.00
PD-L1 CPS ≥ 10	9	2	10	3	45.8%	−0.08
PD-L1 CPS ≥ 20	6	5	9	4	45.8%	−0.04
c-MET H-Score > 50	14	4	4	1	78.3%	0.48
c-MET H-Score > 75	8	7	7	1	65.2%	0.35

T+/LN+: positive in both primary tumor and LN metastasis; T−/LN−: negative in both; T+/LN−: positive in primary tumor only; T−/LN+: positive in *LN* metastasis only. κ: Cohen’s kappa coefficient. Interpretation: <0 less than chance agreement; 0.01–0.20 slight; 0.21–0.40 fair; 0.41–0.60 moderate; 0.61–0.80 substantial; 0.81–1.00 almost perfect agreement.

For PD-L1 expression defined by TPS ≥ 1%, a high concordance rate of 87.5% was observed, largely driven by the high prevalence of PD-L1–positive samples in both compartments. However, Cohen’s kappa was 0.00, indicating no agreement beyond chance due to the near-universal positivity.

Importantly, when applying the CPS ≥ 20 cutoff used to define the high PD-L1 expression subgroup in KEYNOTE-048, concordance decreased markedly to 45.8%. More than half of patients (54.2%, 13/24) would have been classified differently depending on whether the primary tumor or the LN metastasis was analyzed. Specifically, 9 patients (37.5%) exhibited CPS ≥ 20 in the primary tumor but < 20 in the LN metastasis, resulting in different biomarker classifications between both compartments when applying commonly used CPS thresholds. Conversely, 4 patients (16.7%) showed CPS ≥ 20 exclusively in the LN metastasis. Cohen’s kappa was − 0.04, indicating agreement worse than expected by chance.

For c-MET expression (H-Score > 75), concordance was moderate at 65.2%, corresponding to fair agreement (κ = 0.35). Seven patients (30.4%) demonstrated c-MET overexpression in the primary tumor but low expression in LN metastases, whereas only one patient showed the opposite pattern.

Collectively, these findings demonstrate substantial biomarker discordance between primary tumors and matched lymph node metastases. While this observation supports the existence of spatial biomarker heterogeneity in OSCC, the present study was not designed to evaluate the predictive value of biomarker assessment in recurrent or metastatic disease. Therefore, these findings should not be interpreted as evidence for changing current diagnostic workflows but rather as a rationale for future prospective studies investigating compartment-specific biomarker assessment.

### Exploratory outcome analysis

Over a median follow-up of 13.2 years (reverse Kaplan–Meier), 8 of 59 patients (13.6%) developed locoregional recurrence, with a median time to recurrence of 16 months; all recurrences occurred within 7 years. Estimated recurrence-free survival was 94.6%, 91.0%, and 89.2% at 1, 3, and 5 years (Suppl. Figure 2 A). In exploratory analyses, recurrence was not significantly associated with primary-tumor PD-L1 expression (CPS ≥ 20: log-rank *p* = 0.95; HR 1.04, 95% CI 0.26–4.17) or c-MET expression (H-score > 75: *p* = 0.49; HR 2.05, 95% CI 0.25–16.70), and marker expression levels did not differ significantly between patients with and without recurrence (all *p* ≥ 0.37). Primary tumor–lymph node biomarker discordance was likewise not significantly associated with recurrence, for either PD-L1 CPS ≥ 20 (recurrence in 2/13 discordant vs. 2/11 concordant patients; *p* = 0.80; Suppl. Figure 2B) or c-MET H-score > 75 (2/8 vs. 2/15; *p* = 0.47; Suppl. Figure 2 C). These analyses were constrained by the small number of events: even nodal status, an established prognostic factor, was not significantly associated with recurrence (N + vs. N0: *p* = 0.58; HR 1.48, 95% CI 0.37–5.92), consistent with the cohort being underpowered for outcome analysis. Results are summarized in Supplementary Table 2.

## Discussion

In this retrospective study, we analyzed PD-L1 and c-MET expression in primary tumors and matched lymph node metastases of OSCC patients to characterize spatial biomarker heterogeneity between both tumor compartments. Within the limitations of this exploratory study, we observed a significant downregulation of PD-L1 and c-MET expression in lymph node metastases compared with matched primary tumors. This pattern was consistently observed across multiple scoring approaches, including PD-L1 TPS, PD-L1 CPS, and c-MET H-score, and was confirmed in paired analyses. Collectively, these findings indicate that biomarker expression may differ substantially between primary tumors and synchronous metastatic sites, highlighting compartment-specific expression patterns and the biological complexity of spatial heterogeneity in OSCC.

Given the retrospective design and the limited number of matched primary tumor–lymph node metastasis pairs, our findings should be considered exploratory and hypothesis-generating. Although significant differences between tumor compartments were observed, the present study was not designed to determine whether these findings should directly influence diagnostic or therapeutic decision-making.

Based on our previous findings demonstrating that activation of the HGF-MET signaling pathway can promote PD-L1 expression in head and neck squamous cell carcinoma, particularly in metastatic settings [9], we sought to investigate whether PD-L1 and c-MET expression are correlated in primary tumors and their metastases. While experimental data, including studies from our group and others, support a functional interaction between c-MET signaling and immune evasion ([9]; [8, 12]), our clinical data do not demonstrate a statistically significant correlation between these markers, which suggests that these experimentally observed associations may not be readily detectable in a small, underpowered clinical cohort and warrant re-examination in larger studies.

In primary tumors, PD-L1 and c-MET expression appeared largely independent. In lymph node metastases, we observed a trend toward coordinated expression; however, this association did not reach statistical significance and should therefore be interpreted cautiously. These findings suggest that interactions between oncogenic signaling and immune modulation may be context-dependent and become particularly relevant in metastatic lesions. While a consistent co-regulation of PD-L1 and c-MET could not be established, the compartment-specific expression patterns observed in lymph node metastases underscore the potential importance of both pathways in metastatic OSCC. Notably, PD-L1 expression in primary tumors was significantly associated with nodal metastasis (N+), with all N+ patients demonstrating PD-L1 positivity (TPS ≥ 1%) compared to 79.4% of N0 patients. In contrast, c-MET expression showed no significant association with nodal status. This differential association suggests that PD-L1-mediated immune evasion, rather than c-MET-driven invasive capacity, may be more closely linked to the establishment of nodal metastases in OSCC.

Importantly, this association was derived from unadjusted analyses and should not be interpreted as evidence that PD-L1 expression independently predicts nodal metastasis. Potential confounding by tumor stage, grade, lymphovascular invasion, perineural invasion, tumor site, and differences in the local inflammatory microenvironment cannot be excluded. Therefore, these findings should be considered exploratory and hypothesis-generating and require validation in larger cohorts with appropriate multivariable analyses.

Our results further demonstrate a rather compartment-specific expression profile. PD-L1 expression was predominantly weak to moderate in both primary tumors and lymph node metastases, whereas c-MET expression was generally moderate to strong, with the highest levels detected in primary tumors. This pattern may be consistent with the notion that c-MET partly reflects tumor cell-intrinsic biology, whereas PD-L1 expression may be more strongly influenced by dynamic tumor–immune interactions [13]. The observed downregulation of both markers in lymph node metastases may reflect clonal selection, immune editing, or adaptive responses to the distinct immunological milieu of the lymphatic compartment. However, previous reports demonstrated heterogeneous and often conflicting prognostic effects of PD-L1 and c-MET in OSCC, despite these biologically relevant observations [14, 15]. Methodological differences, including the use of CPS, which incorporates both tumor and immune cells, versus TPS, which assesses tumor cells only, may further contribute to inconsistent results across studies [16]. In addition, the retrospective single-center design and the ROI-based assessment of FFPE tissue sections may not fully capture the full extent of intratumoral heterogeneity. Recent spatial profiling studies have demonstrated pronounced cellular and microenvironmental heterogeneity within OSCC, supporting the notion that region-based sampling may incompletely reflect the biological complexity of individual tumors [17].

Although five representative regions were evaluated per specimen, spatial variation in PD-L1 and c-MET expression within individual tumors cannot be excluded.

A limitation of the present study is the historical nature of the cohort. Patients were treated between 2009 and 2011, prior to the introduction of immune checkpoint inhibitors and the routine clinical implementation of PD-L1 testing in HNSCC. Consequently, while the cohort remains suitable for investigating biological differences in biomarker expression between primary tumors and matched lymph node metastases, direct translation of these findings to contemporary biomarker-guided treatment strategies should be interpreted with caution. Validation in modern prospective cohorts treated according to current standards of care will be required.

In addition to the historical nature of the cohort, the PD-L1 assay used in the present study should also be considered when interpreting the clinical relevance of our findings. PD-L1 expression was assessed using clone 28 − 8, whereas pembrolizumab-based treatment decisions in HNSCC are currently based on the 22C3 pharmDx assay [3]. Although previous studies have demonstrated substantial concordance between the 28 − 8 and 22C3 assays, direct extrapolation of 28-8-derived CPS classifications to KEYNOTE-048 treatment categories should be interpreted with caution.

In addition, CPS values were analyzed in their uncapped form to preserve quantitative information across the full range of PD-L1 expression. While this approach differs from routine clinical reporting, where CPS values are commonly capped at 100, it does not affect the categorical analyses based on established CPS thresholds (≥ 1, ≥ 10, and ≥ 20) used in the present study.

In the present study, CPS thresholds were primarily applied to investigate spatial biomarker heterogeneity and biomarker discordance between tumor compartments rather than to determine treatment eligibility.

From a diagnostic pathology perspective, our findings highlight the presence of substantial spatial biomarker heterogeneity between primary tumors and synchronous lymph node metastases in OSCC. While the clinical implications of these findings remain to be established, the observed discordance at commonly used CPS thresholds suggests that biomarker classification may vary across tumor compartments. Future studies including recurrent and distant metastatic lesions are needed to determine the relevance of these observations for biomarker-guided treatment strategies.

Interpretation of c-MET expression warrants particular caution, as clinically validated cutoff values for c-MET are currently lacking in OSCC. Indeed, the definition of c-MET overexpression remains a matter of ongoing debate across HNSCC studies, with substantial heterogeneity in the scoring systems and thresholds used to classify tumors as c-MET–high or c-MET–low. Previous investigations have employed a wide range of immunohistochemical assessment methods and cutoff values, limiting direct comparability between studies. In contrast to the clinically established PD-L1 CPS thresholds, the H-score categories applied in the present study (> 50, > 75, and > 100) were therefore used for exploratory stratification and assessment of biomarker concordance between tumor compartments. Consequently, these categorizations should not be interpreted as clinically validated classifications but rather as a framework for investigating spatial heterogeneity. Future studies are needed to establish biologically and clinically meaningful c-MET thresholds in OSCC.

Several studies have highlighted this issue and reported considerable variability in the criteria used to define c-MET positivity, underscoring the current lack of standardization in the field [7, 18].

The substantial spatial heterogeneity observed in our study indicates that biomarker classifications may differ between tumor compartments and highlights the need for further studies evaluating the impact of sampling site on biomarker assessment [19]. This may be especially relevant in the context of emerging perioperative and biomarker-driven treatment strategies. Our data support a more nuanced interpretation of immunohistochemical results and highlight an area for future investigation regarding the potential role of metastatic tissue or multi-site sampling in biomarker assessment.

A further limitation concerns the outcome data. Structured follow-up in this historical cohort was restricted to locoregional recurrence; distant recurrence, vital status, and distant-metastasis staging were not systematically available, precluding conventional disease-free survival, overall survival, and distant-metastasis-free survival analyses. In exploratory recurrence-free survival analyses, neither primary-tumor PD-L1 or c-MET expression nor primary tumor–lymph node biomarker discordance was significantly associated with recurrence. However, with only eight recurrence events these analyses are clearly underpowered — even nodal status did not reach statistical significance — and should be regarded as hypothesis-generating. The prognostic relevance of compartment-specific PD-L1 and c-MET expression and of biomarker discordance therefore remains to be determined in larger prospective cohorts with complete survival and metastasis follow-up.

From a translational perspective, experimental studies suggest interactions between c-MET signaling and immune regulation, including modulation of PD-L1 expression [9, 12]. Although our cohort did not demonstrate a significant correlation between both markers, the observed compartment-specific expression patterns in lymph node metastases warrant further investigation in larger prospective studies. Combination strategies targeting both c-MET and immune checkpoints such as PD-L1 have shown synergistic antitumor effects in other malignancies, including non-small cell lung cancer and hepatocellular carcinoma, leading to improved CD8⁺ T-cell infiltration and enhanced tumor control [20]. While our findings do not directly support therapeutic conclusions, they provide a biological rationale for further exploration of dual-targeting approaches in OSCC in appropriately designed prospective studies.

Importantly, several c-MET–targeting agents are already clinically available or under investigation, underscoring the translational relevance of our findings [21, 22]. Multikinase inhibitors such as cabozantinib and crizotinib, which exhibit activity against c-MET, are approved in other tumor entities [22, 23]. c-MET-directed monoclonal antibodies such as Ficlatuzumab are currently being evaluated in clinical trials in HNSCC [24]. These developments illustrate the growing clinical interest in MET-directed therapeutic strategies and provide a rationale for further investigation in OSCC.

In summary, our study reveals substantial spatial heterogeneity of PD-L1 and c-MET expression in OSCC and reveals compartment-specific biomarker expression patterns between primary tumors and lymph node metastases. The observed discordance between primary tumors and metastatic lymph nodes highlights the biological complexity of biomarker assessment in OSCC. Future prospective studies integrating spatial and longitudinal molecular profiling are warranted to clarify the biological and clinical relevance of these findings and to determine their implications for biomarker-guided strategies in OSCC.

## Supplementary Information

Below is the link to the electronic supplementary material.

Supplementary Material 1 (DOCX 470 KB)

## Data Availability

No datasets were generated or analysed during the current study.
