# Supplementary material for: Exploring spatial heterogeneity of PD-L1 and c-MET expression in oral squamous cell carcinoma
Source: Oral Maxillofac Surg. 2026 Sep 16;30(1):149. doi: 10.1007/s10006-026-01638-1 (PMC13582147; doi:10.1007/s10006-026-01638-1)
Supplement: Supplementary file 1 [file 10006_2026_1638_MOESM1_ESM.docx]

Suppl. Figure 1. Distribution of biomarker expression in primary tumors and lymph node metastases. 
Violin plots with embedded box plots showing the distribution of (A) PD-L1 TPS, (B) PD-L1 CPS, (C) c-MET H-Score, and (D) c-MET percentage of positive cells in matched primary tumor (red) and lymph node metastasis (blue) samples (n=24 for PD-L1, n=23 for c-MET). Violin plots depict the probability density of the data; box plots show median (horizontal line), interquartile range (box), and whiskers extending to 1.5× IQR. Individual data points are overlaid in black. All markers demonstrate a shift toward lower expression values in lymph node metastases, with narrower distribution ranges compared to primary tumors.
A group of graphs showing different types of tumor cells  AI-generated content may be incorrect.

Supplementary Figure 2. Exploratory recurrence-free survival (RFS) analyses in oral squamous cell carcinoma (n = 59; 8 recurrence events). 
(A) Overall RFS (Kaplan–Meier estimate with 95% confidence band); RFS was 94.6%, 91.0%, and 89.2% at 1, 3, and 5 years, and the median was not reached over a median follow-up of 13.2 years. (B) RFS stratified by primary tumor–lymph node discordance of PD-L1 CPS ≥20 status (paired subgroup, n = 24). (C) RFS stratified by primary tumor–lymph node discordance of c-MET H-score >75 status (paired subgroup, n = 23). Discordance denotes a differing biomarker category between the primary tumor and the matched lymph node metastasis. Tick marks indicate censored patients, and the number of patients at risk is shown below each panel; between-group differences were assessed by the log-rank test. No comparison reached statistical significance, and the small number of events precludes definitive conclusions; these analyses are exploratory and hypothesis-generating.

Suppl. Tables:
Suppl. Table 1. Paired comparison of biomarker expression between primary tumor and lymph node metastases.
Marker
n
Primary Tumor
LN Metastasis
Δ (T–LN)
p-value
PD-L1 TPS (%)
24
27.5
1.0
+16.5
0.003**
PD-L1 CPS
24
45.0
7.5
+17.5
0.026*
c-MET H-Score
23
93.7
70.7
+18.6
0.021*
c-MET % positive
23
73.5
54.8
+15.8
0.086
Wilcoxon signed-rank test. *p<0.05, **p<0.01. Values are medians. Δ represents the median of within-patient paired differences (Primary Tumor minus LN Metastasis), not the difference of medians. Δ(T−LN) positive means primary higher.

Suppl Table 2. Exploratory associations of PD-L1/c-MET status and primary tumor–lymph node discordance with recurrence-free survival in oral squamous cell carcinoma (n = 59; 8 recurrence events).
Variable
n
Recurrence,
positive / discordant
(n/N)
Recurrence,
negative / concordant
(n/N)
Univariable HR
(95% CI)
Log-rank
p
Nodal status — N+ vs N0
59
4/25
4/34
1.48 (0.37–5.92)
0.58
Primary PD-L1 TPS ≥1% vs <1%
59
8/52
0/7
NE†
0.31
Primary PD-L1 CPS ≥1 vs <1
59
8/52
0/7
NE†
0.31
Primary PD-L1 CPS ≥20 vs <20
59
4/29
4/30
1.04 (0.26–4.17)
0.95
Primary c-MET H-score >75 vs ≤75
59
7/45
1/14
2.05 (0.25–16.70)
0.49
PD-L1 CPS ≥20 discordance‡ (T vs LN)
24
2/13
2/11
0.78 (0.11–5.52)
0.80
c-MET H-score >75 discordance‡ (T vs LN)
23
2/8
2/15
2.03 (0.29–14.46)
0.47
Recurrence is shown as events/patients per group; HR from univariable Cox proportional-hazards regression. †NE, not estimable (complete separation: all recurrences occurred in marker-positive patients). ‡Discordance = differing biomarker category between the primary tumor (T) and the matched lymph node (LN) metastasis (paired subgroup). All analyses are exploratory and underpowered; no adjustment for multiple comparisons was applied. CI, confidence interval; CPS, Combined Positive Score; HR, hazard ratio; LN, lymph node metastasis; T, primary tumor; TPS, Tumor Proportion Score.
